# Anthropometric Differences in Community- Versus Clinic-Recruited Infants Participating in a Trial of Azithromycin for Prevention of Childhood Mortality in Burkina Faso

**DOI:** 10.4269/ajtmh.23-0298

**Published:** 2023-10-02

**Authors:** Mamadou Ouattara, Ali Sié, Mamadou Bountogo, Valentin Boudo, Clarisse Dah, Elodie Lebas, Huiyu Hu, Travis C. Porco, Benjamin F. Arnold, Thomas M. Lietman, Catherine E. Oldenburg

**Affiliations:** ^1^ Centre de Recherche en Santé de Nouna, Burkina Faso, Nouna;; ^2^ Francis I Proctor Foundation, University of California, San Francisco, California;; ^3^ Department of Epidemiology & Biostatistics, University of California, San Francisco, California;; ^4^ Department of Ophthalmology, University of California, San Francisco, California

## Abstract

Clinic-based recruitment for preventative interventions for child health may select for healthier populations compared with community-based outreach. Nutritional status during infancy as measured by anthropometry is predictive of mortality, growth faltering later in life, and poor cognitive development outcomes. We evaluated baseline differences in infant nutritional status among children recruited directly in their community versus clinic recruitment among infants participating in a trial of azithromycin compared with placebo for prevention of mortality in three districts of Burkina Faso. Infants between 5 and 12 weeks of age were recruited in their community of residence via vaccine outreach teams or in primary health-care clinics during vaccine clinics. Weight, height, and mid upper arm circumference were measured. We used linear and logistic regression models to compare anthropometric outcomes among community and clinic recruited infants, adjusting for age at enrollment, gender, and season. Among 32,877 infants enrolled in the trial, 21,273 (64.7%) were recruited via community outreach. Mean weight-for-age z-score (WAZ) was −0.60 ± 1.2 (SD), weight-for-length z-score (WLZ) was −0.16 ± 1.5, and length-for-age z-score was−0.53 ± 1.3. Infants enrolled in the community had lower WAZ (mean difference, −0.12; 95% CI, −0.20 to −0.04) and WLZ (mean difference, −0.21; 95% CI, −0.32 to −0.09). Community-recruited infants were more often underweight (WAZ < −2; odds ratio [OR], 1.25; 95% CI, 1.09–1.43) and wasted (WLZ < −2; OR, 1.54; 95% CI, 1.31–1.79). There was no evidence of a difference in height-based measures. Community and clinic recruitment likely reach different populations of children.

## INTRODUCTION

Provision of pharmaceutical and nonpharmaceutical interventions directly in communities is a common strategy for health-care delivery in settings with reduced access to health care in many areas of sub-Saharan Africa. For child health, examples of these interventions include distribution of seasonal malaria chemoprevention during the high malaria transmission season, community vaccine campaigns, and community health worker-led screening for acute malnutrition.^1^^–^^3^ Community-based delivery strategies have several advantages over clinic-based delivery because they do not require individuals receiving care to travel potentially long distances to seek care. As such, children reached via community-based delivery outreach may differ from those receiving services in clinic-based settings.

Twice-yearly community-based distribution of azithromycin has been shown to reduce all-cause childhood mortality significantly among children 1 to 59 months of age.^4^ Subsequent work evaluating azithromycin in clinic-based settings has generally not found an effect on outcomes in children recruited via clinics.^5^^–^^7^ There are several possible reasons for this discrepancy. Treatment of an entire community at the same time may interrupt, in part, transmission of pathogens that leads to childhood mortality. Azithromycin targeted to individuals likely would not have the same effect as mass distribution.^8^ In addition, community-based distribution strategies reach children who have reduced access to clinics and who are therefore missed by clinic-based enrollment strategies.

Burkina Faso is a landlocked country in West Africa that experiences seasonal malaria transmission and has a relatively high burden of childhood undernutrition. In a population-based sample of infants in Nouna District, Burkina Faso, approximately 20% of infants 1 to 11 months of age were underweight.^9^ Previous work has shown that children who live farther from health-care facilities or clinics are less likely to receive care in primary health-care facilities in Burkina Faso compared with those who live closer to facilities.^10^ These children may be more vulnerable and have worse outcomes compared with those enrolled in clinic-based settings, and recruitment of children in clinics may select for overall healthier populations or populations already engaged in care who do not benefit from additional antibiotic-based intervention. Understanding differences in indicators of child health between populations may also be beneficial for targeting interventions. For example, interventions could be targeted via recruitment methods that identify the most vulnerable children.

Here, we used data from an individually randomized trial of azithromycin for prevention of infant mortality^11^ delivered during routine childhood vaccination that enrolled infants in both community (via vaccine outreach directly to communities) and clinic (via routine clinic-based vaccination) settings to evaluate differences in anthropometric measurements, including underweight, stunting, and wasting, between children recruited in community settings compared with clinic settings. Childhood undernutrition has been linked to increased risk of mortality and worse longer term developmental outcomes.^12^^–^^16^ We hypothesized that infants recruited in community settings have worse anthropometric measures compared with those in clinic settings, reflecting a more vulnerable population.

## MATERIALS AND METHODS

### Parent study overview.

Data for this secondary analysis arose from an individually randomized trial of single-dose oral azithromycin compared with placebo administered at age 5 to 12 weeks in conjunction with routine vaccination for prevention of infant mortality by 6 months of age.^11^ Complete methods for the parent study have been reported previously.^11^

### Study setting.

This study was conducted in Kossi, Banfora, and Karankasso-Vigue provinces of Burkina Faso between September 2019 and September 2022. Burkina Faso is located in the Sahel, and experiences highly seasonal rainfall from approximately July through October. In the study area, regular outreach is made to each community for childhood vaccination. Each month, a vaccination team consisting of nurses from primary health-care facilities performs outreach directly in communities to offer childhood vaccinations.^17^ Vaccinations are also provided at vaccination clinics at local primary health-care facilities (Centers de Santé et de Promotion Sociale). For participants recruited in Kossi Province, GPS data were available for both the community of residence of the infant as well as the primary health-care facility used by their community, as the communities participating in the study in Kossi were enrolled in a cluster randomized trial in which GPS data were collected.^11^ These data were not available for infants enrolled outside of Kossi Province.

### Participants and recruitment.

Infants were eligible for the parent trial if they were between 5 and 12 weeks of age at the time of enrollment, had no documented allergy to macrolides, and their family was planning to stay in the study area for the duration of the study (6 months). Participants were recruited either directly in their communities as part of vaccine outreach visits or in the primary health-care clinic during clinic vaccinations. The location of recruitment and enrollment of each infant enrolled in the trial was recorded in the study’s mobile data collection application. Infants who were recruited in the community but referred to and enrolled in the clinic were considered community recruited.

### Anthropometric measurements.

At baseline, weight, length, and mid upper arm circumference (MUAC) were collected on all infants. Weight was measured using a standard digital infant scale. A single weight measurement was acquired. Length was measured using a ShorrBoard (Weight and Measure LLC, Olney, MD). Length measurements were acquired in triplicate, with the median used for analysis. Mid upper arm circumference was measured using a standard MUAC tape. Weight-for-age z-score (WAZ), weight-for-length z-score (WLZ), and length-for-age z-score (LAZ) were calculated according to the WHO 2006 growth standards.^18^ Anthropometrists underwent didactic and practical training on measuring weight, height, and MUAC to standardize measurements across anthropometrists prior to the start of the study. Regular supervision to ensure fidelity to the anthropometry protocol was undertaken, with retraining as necessary.

### Statistical analyses.

Descriptive statistics were calculated among those recruited in the community and clinic using medians and interquartile ranges for continuous variables, and proportions for categorical variables. To assess the relationship between community versus clinic enrollment and continuous anthropometric measures (weight, height, WAZ, WLZ, LAZ, and MUAC), we constructed a series of linear regression models, including unadjusted; adjusted for age, gender, and month of enrollment; and adjusted for age, gender, month of enrollment, and distance from the child’s community of residence and the health-care facility. Because GPS coordinate data were only available for infants recruited in Kossi Province, models adjusting for distance from the community of residence to the clinic were restricted only to infants in Kossi Province. We evaluated the relationship between community versus clinic recruitment and dichotomized measures of anthropometric status, including underweight (WAZ < −2), wasted (WLZ < −2), and stunted (LAZ < −2) using a similar series of logistic regression models. The SEs of all models were clustered by the community of recruitment. Because missing data were extremely rare (< 0.1%), complete case analysis was used. All analyses were done using Stata 17.0 (StataCorp, LLC, College Station, TX).

## RESULTS

Among 32,877 infants enrolled in the trial, 21,273 (64.7%) were recruited directly in their community and 11,604 (35.3%) were recruited in clinics. Infants recruited in the community tended to be younger than those recruited in clinics (median age, 44 days versus 50 days). As expected, infants recruited in the community lived considerably farther from the clinic than those recruited in clinics (median distance from the community of residence to the clinic, 4.7 km versus 1.5 km; Table 1). The distribution of infant’s sex and season of enrollment was similar between the two groups (Table 1).

**Table 1:** Baseline demographic characteristics between children recruited in community settings vs. clinic settings

Characteristic	Community	Clinic
*N*	21,273	11,604
Females, *n* (%)	10,446 (49.1)	5,736 (49.4)
Age, days; median (IQR)	44 (35–59)	50 (37–64)
Enrolled in rainy season (July–October), *n* (%)	7,294 (34.3)	3,985 (34.3)
Distance to health center, km; median (IQR)*	4.7 (1.9–6.5)	1.5 (0.6–4.2)

IQR = interquartile range.

* Distance from village to health center available for 23,768 enrolled infants.

In the full cohort, in models adjusted for the infant’s age, gender, and season of enrollment, weight-based measures of nutritional status, including WAZ and WLZ, were significantly less in infants recruited in their community of residence compared with those recruited in the clinic (Table 2). There was no evidence of a difference in length or LAZ in community-recruited infants compared with those recruited from clinics (Table 2). These results were attenuated when adjusting for distance from the community to the clinic; WAZ and WLZ were no longer statistically significantly different between community- and clinic-recruited infants.

**Table 2:** Baseline anthropometric differences between infants recruited in community settings vs. clinic settings

Outcome	Community, mean (SD)	Clinic, mean (SD)	Unadjusted (95% CI)*	Age, gender, and season adjusted (95% CI)†	Distance adjusted (95% CI)‡
Weight, kg	4.6 (0.9)	4.7 (0.9)	−0.17 (−0.28 to −0.06)	−0.08 (−0.13 to −0.02)	−0.02 (−0.06 to 0.02)
Height, cm	55.2 (3.1)	55.5 (3.1)	−0.31 (−0.64 to 0.06)	0.02 (−0.17 to 0.22)	0.09 (−0.12 to 0.30)
WAZ	−0.65 (1.2)	−0.52 (1.2)	−0.12 (−0.21 to −0.04)	−0.12 (−0.20 to −0.04)	−0.04 (−0.11 to 0.03)
WLZ	−0.25 (1.5)	−0.01 (1.4)	−0.23 (−0.35 to −0.11)	−0.21 (−0.32 to −0.09)	−0.13 (−0.27 to 0.01)
LAZ	−0.52 (1.3)	−0.55 (1.3)	0.03 (−0.07 to 0.12)	0.02 (−0.08 to 0.12)	0.05 (−0.06 to 0.15)
MUAC, cm	12.1 (1.2)	12.1 (1.2)	−0.06 (−0.23 to 0.10)	0.03 (−0.09 to 0.15)	0.08 (−0.009 to 0.17)

LAZ = length-for-age z-score; MUAC = mid upper arm circumference; WAZ = weight-for-age z-score; WLZ = weight-for-length z-score.

* Unadjusted linear regression model.

† Linear regression model adjusted for infant age, gender, and season of enrollment.

‡ Linear regression model adjusted for infant age and gender, and distance from their community of residence to the primary health-care facility (restricted to 23,768 infants enrolled in Kossi Province).

The prevalence of both underweight (defined as WAZ < −2) and wasting (WLZ < −2) was significantly greater in infants recruited in the community compared with the clinic, and the relationship between recruitment location was stronger with wasting (adjusted odds ratio [aOR], 1.55; 95% CI, 1.34–1.84) compared with underweight (aOR, 1.25; 95% CI, 1.20–1.43; Table 3). Odds ratios for both underweight and wasting were attenuated in models adjusted for distance from the community of residence to the clinic, but recruitment location remained associated statistically significantly with wasting (aOR, 1.40; 95% CI, 1.18–1.67).

**Table 3:** Probability of underweight, wasting, and stunting among infants recruited in community settings vs. clinic settings

Outcome	Community, *n* (%)	Clinic, *n* (%)	Unadjusted (95% CI)*	Age, gender, and season adjusted (95% CI)†	Distance adjusted (95% CI)‡
Underweight§	2,599 (12.2)	1,169 (10.1)	1.24 (1.09–1.42)	1.25 (1.09–1.43)	1.12 (0.98–1.27)
Wastedǁ	2,286 (10.8)	833 (7.2)	1.55 (1.34–1.84)	1.54 (1.31–1.79)	1.40 (1.18–1.67)
Stunted¶	2,387 (11.2)	1,368 (11.8)	0.94 (0.80–1.12)	0.99 (0.82–1.18)	0.95 (0.78–1.15)

* Unadjusted logistic regression model.

† Logistic regression model adjusted for infant age, gender, and season of enrollment.

‡ Logistic regression model adjusted for infant age and gender, and distance from their community of residence to the primary health-care facility (restricted to 23,768 infants enrolled in Kossi Province).

§ Underweight is defined as weight-for-age Z-score (WAZ) < −2.

ǁ Wasting is defined as weight-for-length Z-score (WLZ) < −2.

¶ Stunting is defined as length-for-age Z-score (LAZ) < −2.

## DISCUSSION

We found evidence that infants recruited via community outreach efforts were, on average, at a greater risk of weight-based anthropometric deficits compared with infants recruited via clinic visits. Community-based outreach for public health interventions has the potential to reach individuals who do not have access to health-care facilities. Previous work has demonstrated a distance decay effect in health-care use, where people who live farther from health-care facilities are less likely to seek care compared with those who live closer.^10^^,^^19^ Recruitment of children from clinic-based populations likely selects for an overall healthier population. These children may have reduced barriers for accessing health care (including distance and time-related barriers for care seeking), and may have benefited from earlier and more frequent contact with the health-care system. For example, delays in seeking care for malaria may mean that children with better access to health-care facilities have better outcomes, and those who live closer to health-care facilities may be more likely to be up to date on vaccinations and other preventative health interventions. Although some evidence has suggested variations in anthropometry, and access to food insecurity is seasonal in the study area, previous work in this study area has not indicated a difference in infant underweight by season.^9^ Models adjusting for season did not affect results demonstrably, and a similar proportion of infants recruited in the community and the clinic were recruited in the rainy season compared with the dry season. This suggests that season is not a primary explanation for differences observed in this analysis. Overall, these results suggest that direct outreach to communities reaches a different population of children compared with relying on clinic-based recruitment.

Equity-based approaches for delivery of health care have considered direct delivery of interventions in communities that are difficult to reach as a result of geography or other barriers. In Niger, distance-based vaccine and other health-care delivery strategies are being implemented to deliver health care to children in the hardest to reach communities, such as mobile vaccination and health-care provision in communities that are more than 15 km from a health facility.^20^ Similar outreach strategies are used in Burkina Faso. In this analysis, community-recruited infants lived in communities that were, on average, 3 km farther from the health clinic compared with those recruited in clinics. This is consistent with greater outreach activities in communities that are farther from health facilities. In the subset of children for whom GPS coordinate data were available, adjusting for distance from the community to the clinic attenuated differences in anthropometric measures between community- and clinic-recruited infants. This suggests that infants who are harder to reach have worse anthropometric deficits, and that community-based recruitment can reach these infants effectively.

We did not find evidence of a difference in height-based anthropometric measures, including LAZ and stunting. Stunting is typically considered to be a result of chronic malnutrition, although the greatest incidence of stunting occurs between birth and 3 months,^21^ and often is the result of intrauterine growth restriction and preterm birth, which are influenced by maternal characteristics such as maternal age and undernutrition, and infection during pregnancy.^22^^,^^23^ We did not have data on maternal characteristics in this study, and are unable to comment on whether maternal characteristics differed for mothers of infants recruited in communities versus clinics. Similarly, we did not have data on breastfeeding or dietary status for mothers or infants recruited in this study, which influence weight-based measurements at enrollment. Wasting is often more acute than stunting, and these differences may reflect postpartum changes in infants independent of maternal factors. Studies including maternal characteristics are needed to evaluate this hypothesis.

These results have implications for trials of child health interventions. Results of trials relying on clinic-based recruitment are likely not generalizable to all children in the general population. Children who are recruited via well-child visits (e.g., vaccination) are likely healthier on average than the general population. Although randomized controlled trials of azithromycin for mortality have been underpowered for subgroup analysis, there has been some evidence that nutritional status may modify the effect of azithromycin on infant mortality.^24^ Differences in recruited populations could have power implications for trials (for example, if healthier children are less likely to have the primary outcome of interest in a trial). An example of this is the NAITRE study,^25^ a randomized controlled trial evaluating single-dose azithromycin compared with placebo during the neonatal period for prevention of infant mortality. The trial enrolled exclusively in clinics that were close to tertiary care facilities for diagnosis and treatment of infantile hypertrophic pyloric stenosis, a potential complication of neonatal exposure to macrolides.^26^ The mortality rate in the trial was considerably less than anticipated, resulting in the trial having less power than anticipated.^5^ Taking interventions directly to the community, although more burdensome logistically, is a more equitable approach to delivery of public health interventions and may be required to reach the most vulnerable children most in need of life-saving interventions.

These results must be considered in the context of several limitations. All infants enrolled in the trial were reached by vaccine outreach teams or in clinics. It is possible that infants who are not reached at all by vaccination teams are substantively different than those who are. The number of “zero-dose” children, or those who have had no doses of a given vaccine series, varies substantially by country and is low in Burkina Faso.^27^ Strategies for reaching children who are missed by vaccine outreach are highly context specific. Although the same size was large, infants enrolled in this trial are not a random sample of the general population. And although our study’s inclusion criteria were fairly broad, these infants were enrolled in a randomized controlled trial, and there may be substantive differences between study participants and infants in the general population. We had little covariate data available related to socioeconomic status, dietary diversity, maternal characteristics, patterns of infection (e.g., malaria), health-seeking behavior, and other factors. These factors may differ for children recruited in communities and clinics, and may also be related to weight, and thus may explain some of the differences observed here. However, the primary goal of this analysis was to describe differences in community- and clinic-recruited populations rather than causal inference. Although having these data may help explain differences, they do not affect the ultimate conclusions about differences in the trial populations.

In this study of trial-enrolled infants, infants recruited via community-based vaccine outreach had lower anthropometric scores for most weight-related measures, reflecting a worse overall nutritional status. We posit that these children may be more vulnerable to poor child health outcomes, including increased risk of mortality, chronic undernutrition, and poor child development outcomes. Studies recruiting children exclusively from clinics will likely miss important subgroups that are more vulnerable. As a result, such studies may not be generalizable to populations that have reduced access to health-care facilities.
